# Heavy metal mediated phytotoxic impact on winter wheat: oxidative stress and microbial management of toxicity by *Bacillus subtilis* BM2

**DOI:** 10.1039/c9ra00333a

**Published:** 2019-02-19

**Authors:** Asfa Rizvi, Bilal Ahmed, Almas Zaidi, Mohd. Saghir Khan

**Affiliations:** Department of Agricultural Microbiology, Faculty of Agricultural Sciences, Aligarh Muslim University Aligarh 202002 Uttar Pradesh India asfarizvi09@gmail.com

## Abstract

Heavy metals are toxic environmental contaminants, which severely affect microbial composition and functions and, concurrently, crop production. Due to these issues, the present study focussed on the selection of metal tolerant microbes endowed with metal detoxification abilities and their role in the management and remediation of metal contaminated soils. The metal tolerant bacterium BM2, identified as *Bacillus subtilis* by 16SrRNA gene sequencing, survived well under metal pressure and tolerated 1600 and 2000 μg mL^−1^ of Ni and Pb, respectively. The inhibitory impact of metals on wheat increased consistently with a progressive increase in metal concentration. Deposition of Ni and Pb within root and leaf and oxidative stress were validated by SEM, EDX and CLSM. The overall growth parameters of wheat grown under metal stress were improved following *B. subtilis* BM2 colonization. As an example, *B. subtilis* with 195 mg Pb kg^−1^ enhanced the length and dry biomass of shoots by 14% and 23%, respectively, over the control. Also, strain BM2 improved the grain yield significantly by 49% at 870 mg Ni kg^−1^ and by 50% at 585 mg Pb kg^−1^ compared to uninoculated plants. Moreover, *B. subtilis* BM2 relieved the metal stress on wheat and caused a significant drop in proline and malondialdehyde content and the activities of antioxidant enzymes, like catalase (CAT), superoxide dismutase (SOD) and glutathione reductase (GR)*.* This study, therefore, provided solutions to the metal toxicity problems faced by winter wheat and clearly suggests that the metal detoxification potential of *B. subtilis* BM2 could be greatly useful in the management of metal polluted soils.

## Abstract 2

The bacterial management of heavy metal toxicity to durum wheat under a metal stressed environment was demonstrated.

## Graphical abstract

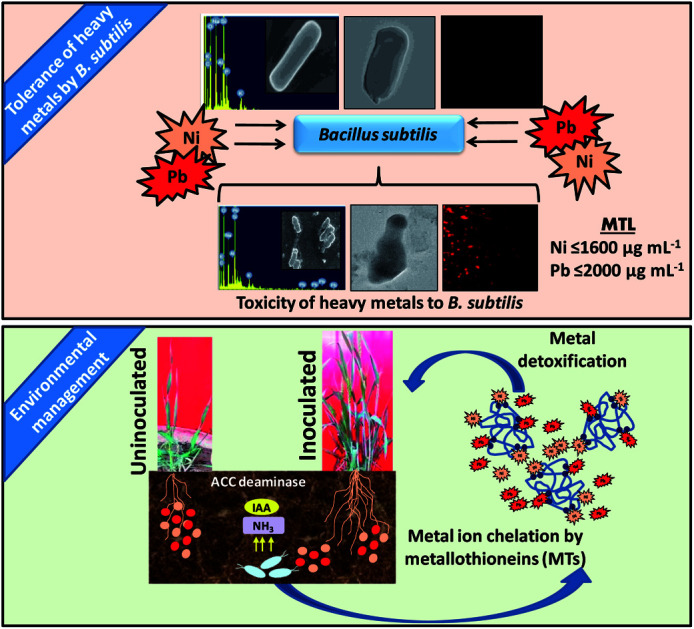

## 1. Introduction

Among various soil pollutants, heavy metal pollution is one of the major global challenges and has attracted the attention of scientists to protect/preserve the very sustainability of natural ecosystems. In many developed and developing countries the heavy metal pollution has risen due to rapid industrialization, long term use of poor quality waters (untreated waste water) for irrigation and intensive agricultural practices. These anthropogenic activities in isolation or simultaneously add considerable amounts of heavy metals to soils.^1,2^ Unfortunately, heavy metals are biologically non-destructible constituents, and hence, they persist indefinitely in soil ecosystems. Once accumulated in soils, heavy metals adversely affect the soil dynamics^3^ and microbial composition and functions,^4^ leading eventually to losses in soil fertility and ultimately crop production.^5,6^ From plants, metals can be introduced into the food chain, which subsequently raises the risk of toxicity to both animals and humans.^7,8^ However, not all metals are inhibitory at any given concentration, but rather their toxicity and deleterious impact differ from species to species and with the concentrations of metals as well as the age and genotypes of plants.^9–11^ Indeed, some metals are essential and play important roles in regulating various metabolic functions of plants. For example, while high concentrations of Ni negatively affect plant growth,^12^ as a trace micronutrient element, it aids in regulating physiology including seed germination and nitrogen metabolism of plants.^13,14^ Some common symptoms associated with Ni phytotoxicity include (i) leaf chlorosis, (ii) abnormal root/shoot growth, (iii) deformity in various plant organs and (iv) altered nutrient uptake. Due to these effects, enhanced accumulation of Ni inside plant tissues results in massive losses in both quantity and nutritive quality of agronomically important and edible crops.^15^ Lead is another bioactive metal which, at elevated levels, decreases cell division in plants and causes brittleness in leaves with the appearance of dark purple spots.^16^ Imbalance in water transport, disturbed composition and uptake of mineral nutrients, interruption of photosynthesis, altered enzymatic activities and overall hormonal imbalance are a few other significant symptoms of Pb toxicity in plants.^17,18^ Apart from the direct inhibitory effect of metals on growing plants, metals can also destroy cells by causing overproduction of reactive oxygen species (ROS), which damage/impair antioxidant defense systems resulting in oxidative stress.^19–21^ Among ROS, the radicals hydroxyl and superoxide and non-radicals H_2_O_2_ and singlet oxygen, for example, have been found to cause critical damage to biological macromolecules, including DNA, proteins and lipids.^22^ Unfortunately, the level of ROS production and consequent cellular toxicity increases rapidly when plants are exposed to heavy metals.

Among various food crops, cereals form an intricate part of the human diet. Wheat (*Triticum aestivum* L.) is one of the most agronomically important cereal crops, cultivated throughout the world and consumed widely as human food. Moreover, various biotic and abiotic factors like heavy metal stress along with salinity, drought, and soil infertility severely affect the growth of wheat plants and consequently limit its production.^23,24^ Hence, the heavy metal pollution of soils requires special and urgent attention in order to restore the fertility of soils and concurrently to enhance the production of wheat, even in metal contaminated soils. In this regard, environment friendly and cost effective approaches like the use of beneficial soil microbiota colonizing the rhizospheres, often termed plant growth promoting rhizobacteria (PGPR), endowed with the unique property of heavy metal tolerance and plant growth promotion, have been suggested as a strategy in the management of derelict soils.^25,26^ However, the results have been conflicting and inconsistent. Nevertheless, metal tolerant microbes when used as a microbial inoculant (biofertilizer) have been reported to detoxify metal toxicity by altering/limiting the bioavailability of metals in soil by several mechanisms including (i) acidification, (ii) chelation of metal ions, (iii) precipitation, (iv) exclusion, (v) metal ion sequestration by the release of organic acids and (vi) transformation of heavy metals from highly toxic to less/non-toxic forms.^27,28^ Besides these, the synthesis and release of metal binding proteins and peptides like metallothioneins (MTs) and phytochelatins (PCs) are some other strategies adopted by metal tolerant bacteria to chelate and sequester metals and concurrently to defend against heavy metal stress.^29^ Therefore, considering the importance of soil contamination, conflicting reports on metal toxicity to wheat, nutritive value of wheat in human dietary systems and the role of PGPR in the effective and viable management of metal polluted soils, the present study was designed to explore the following: (i) recovery and selection of metal tolerant beneficial microbes possessing the ability to secrete active biomolecules supporting wheat growth, (ii) assessment of nickel and lead toxicity to both isolated microbes and wheat plants, (iii) assaying the cellular distribution/localization of nickel and lead through SEM, EDX and CLSM and (iv) to evaluate the bioremediation potential and capability of metal tolerant microbes to facilitate the performance of wheat plants even in metal polluted soils.

## 2. Materials and methods

### 2.1 Metal concentration and microbial composition of experimental soil

A soil sample was collected from the rhizospheres of bajra (*Pennisetum glaucum*) cultivated in fields irrigated with wastewater originating from steel and paint manufacturing industries located at Mathura bypass road, Aligarh (27°53′N 78°05′E 27.88°N 78.08°E), Uttar Pradesh, India (S1) and the concentration of metals was determined according to the method of ref. 30. Conventional soil samples (non-polluted) were also collected from the agricultural fields of Faculty of Agricultural Sciences, Aligarh Muslim University, Aligarh (S2). The soil samples were collected in polythene bags (30 × 20 cm) from two rhizospheric sites (S1 and S2) using a dagger by removing the topsoil up to a depth of 12 cm. For heavy metal analysis, the soil samples were digested in aqua regia (HNO_3_ and HCl in a ratio of 1 : 3) and the heavy metal content was estimated by atomic absorption spectrophotometry (AAS). For isolation of bacteria from metal contaminated soil in the *Pennisetum glaucum* rhizospheres, the soil was serially diluted and bacterial colonies were obtained by spreading 100 μL of soil suspension onto nutrient agar plates when incubated at 28 ± 2 °C overnight. The bacterial colonies that appeared following incubation were picked and re-streaked three times on nutrient agar medium so as to ascertain the purity of the bacterial culture.

### 2.2 Selection and identification of metal tolerant strain

For assessing the metal toleration ability, the isolated bacterial strains were cultured on nutrient agar plates (NAP) amended with increasing concentrations (0–2400 μg mL^−1^; at a two-fold dilution interval) of Ni (NiCl_2_·6H_2_O) and Pb [Pb(CH_3_COO)_2_·3H_2_O]. A spot (10 μL of fully grown bacterial cell suspension) consisting of approximately 10^7^ cells per mL was placed on metal treated NAP. Plates were incubated at 28 ± 2 °C for 48 h and observed for bacterial survivability. The bacterial strains surviving at the highest metal concentration were scored as the metal tolerant strains. Of the total 10 bacterial strains, strain BM2, exhibiting maximum tolerance to Ni and Pb, was chosen for further studies. The selected strain was identified to genus level primarily by morphological and biochemical characteristics while it was identified to species level by a 16S rRNA partial gene sequencing service, Macrogen, Seoul, South Korea, using universal primers, 785F (5′-GGATTAGATACCCTGGTA-3′) and 907R (5′-CCGTCAATTCMTTTRAGTTT-3′). Following a BLASTn search, the processed nucleotide sequence was submitted to the GenBank sequence database. Also, a phylogenetic tree representing the evolutionary relatedness of strain BM2 with other bacterial strains was constructed by the neighbour joining method using MEGA 6.0 software.

### 2.3 Cellular metal distribution and cytotoxic damage to bacterial strain BM2 assessed under SEM, EDX, TEM and CLSM

In order to assess the cellular and morphological damage/anomaly, if any, caused to strain BM2, the strain was cultured in nutrient broth (NB) treated with 200 μg Ni mL^−1^ and 400 μg Pb mL^−1^. To visualize the cytotoxic damage, metal treated bacterial cells were subjected to SEM (model JSM 6510 LV; JEOL, Japan) analysis. For this, 2 mL each of metal treated and untreated bacterial cell suspension was centrifuged (at 10 000 rpm) for 10 min and the cell pellets were washed at regular intervals three times using phosphate buffered saline (PBS) to completely eliminate debris/traces of NB. Then, the washed cell pellets were fixed in primary fixative (2.5% glutaraldehyde and 2% paraformaldehyde) overnight at 4 °C. The pellets were rinsed again with PBS three times at a uniform interval of 5 min each. Lastly, the cell pellets were dehydrated by 30%, 50%, 70%, 90% and 100% ethanol. Then, 2 μL suspensions of the dehydrated pellets were mixed with 8 μL of double distilled water (DDW) on a glass cover slip and observed under SEM. The distribution and percentage of heavy metals inside bacterial cells was determined by EDX spectroscopy. To further validate the disruption caused by heavy metals to the bacterial cell envelope, the suspension of cell pellets was dehydrated with ethanol gradients. Carbon coated copper grids were prepared using 10 μL of bacterial sample and the samples were viewed under TEM (model TEM 2100; JEOL, Japan).

To analyse cell death under metal stress, bacterial cells were grown in NB medium amended with 25, 50, 100 and 200 μg mL^−1^ each of Ni and Pb. NB medium without heavy metals but inoculated with bacterial cells was also run as a control for comparison. Both treated cells and untreated control cells were observed under CLSM (Model LSM-780, Zeiss, Germany). Following centrifugation, the cell pellets were washed three times with PBS for 15 min and then dissolved again in PBS. 100 μL samples of bacterial cell suspension of metal treated and untreated cells were mixed with 10 μL each of propidium iodide (PI) and acridine orange (AO), both of which were prepared in PBS maintained at 1 mg mL^−1^ concentration. The mixture of bacterial cell suspension and fluorescent dyes was then incubated for 10 min at room temperature. The resulting suspension was again centrifuged (at 5000 rpm) for 10 min to remove traces of unbound dye, if any. Next, the cell pellets were re-suspended in 500 μL of PBS and a thin smear of this suspension was prepared on a glass slide. The samples were then scored for PI stained dead cells with excitation/emission maxima of 493/636 nm for PI. Additionally, AO served as a second indicator to differentiate between viable and dead cells. A sample for CLSM observation was prepared in darkness in order to avoid the bleaching of the dyes when exposed to light.

### 2.4 Assessment of metal removal strategy: induction of metallothioneins by bacterial strain BM2

The metallothioneins (MTs) secreted by strain BM2 under heavy metal pressure were quantitatively assessed by the method of ref. 31 with some modifications. The bacterial strain was cultured in NB containing 200 μg Ni mL^−1^ and 400 μg Pb mL^−1^ for 48 h at 28 ± 2 °C on a shaking incubator. A control set (without heavy metals) was also included for comparison. Following incubation, bacterial cells was centrifuged and cell pellets were obtained. The pellets were suspended in lysis buffer with a formulation of 0.5 M sucrose prepared in 20 mM Tris–HCl buffer (pH = 8.6) containing 0.01% mercaptoethanol. Pellets were sonicated for 2 min using a Branson Digital Sonifier (Branson Ultrasonics Corporation, Danbury, USA) in order to completely lyse the cells. The sonicated sample was centrifuged again and the supernatant was separated from the pellets. To 1 mL of supernatant, 1.05 mL of cold absolute ethanol (chilled at −30 °C) and 80 μL of CHCl_3_ (chloroform) were added. The samples were centrifuged (under cold conditions) at 10 000 rpm for 10 min with three volumes of cold ethanol added to the supernatant and the suspension was chilled at −30 °C for 1 h. The samples were centrifuged at 10 000 rpm for 10 min and the cell pellets were washed with a mixture of ethanol, chloroform and homogenization buffer in the ratio 87 : 1 : 12 μL and again centrifuged at 10 000 rpm for 10 min. The pellets were dried to complete evaporation and re-suspension of the dried pellets was done in 300 μL of 5 mM Tris–HCl and 1 mM EDTA (pH = 7). The MTs fraction so obtained was mixed with 4.2 mL of 0.43 mM 5,5′-dithio-bis-nitrobenzoic acid (Ellman's reagent) in 0.2 M phosphate buffer (pH = 8) and kept at room temperature for 30 min for the reaction to occur. The reduced sulfhydryl concentration in the samples was recorded at 412 nm and the MTs in each sample were quantified against a standard curve of reduced glutathione (GSH). The MT content in the samples was calculated using the standard curve equation of ref. 32.

### 2.5 Morphological distortions in wheat plant organs: metal localization/distribution and cellular damage to wheat roots observed under CLSM

The structural alteration in the roots and foliage of wheat plants grown under Ni and Pb pressure was revealed under SEM, EDX and CLSM. The elemental composition and metal distribution in the roots and leaves of wheat plants were detected by EDX. For toxicity studies, wheat plants were grown on soft agar plates amended with 100 μg mL^−1^ of Ni and 200 μg mL^−1^ of Pb. An untreated control was also run in parallel for comparison. The alterations in roots and foliage morphology were visualized under SEM. Also, the composition of elements and distribution of heavy metals within the plant organs were detected by capturing the EDX spectra along with elemental mapping of particular regions of the metal treated and untreated roots and foliage of wheat plants. Moreover, the roots detached from plants treated with 25, 50, 100, 200 and 400 μg mL^−1^ each of Ni and Pb were scanned for live and dead cells using AO and PI fluorescent dyes and observed under an LSM-780 Confocal Microscope (Zeiss, Germany).

### 2.6 Metal toxicity to wheat plants and remediation of toxicity by bioinoculant BM2

For assessing the response of wheat plants to metals, uniform and healthy seeds of wheat (cv. 1055) were surface sterilized with 4% sodium hypochlorite for 3 min and the seeds were then washed with 6–7 changes of sterile water. The sterilized seeds were dipped in overnight grown liquid culture of metal tolerant strain BM2 for 2 h using 1% guar gum powder as adhesive to deliver approximately 10^8^ cells per seed. Also, the non-bacterized seeds, soaked in sterile water only, served as control. The bioprimed and uninoculated seeds (5 seeds per pot) were sown in earthen pots (22 cm high and 25 cm internal diameter) having 4 kg unsterilized alluvial sandy clay loam soil. Two days prior to sowing of seeds, Ni and Pb at normal (1×), double (2×) and three times (3×) the normal dose were added to the experimental clay pots. The normal dose of Ni and Pb was 290 and 195 mg kg^−1^ soil, respectively. The 1× concentration of Ni and Pb refers to the normal concentration from here onwards throughout the manuscript, and these doses were added to the experimental soil. These doses of Ni and Pb were chosen for the pot trials of wheat in order to reproduce the concentrations that had been detected in the surveyed soil samples. There were 14 treatments with three replications each. Pots were arranged in a completely randomized block design. Fifteen days after emergence, thinning of plants was done and only two plants per pot were maintained till harvest. The pots were watered regularly with tap water and were kept in open field conditions. The position of the pots was changed regularly so as to avoid any positional effects on wheat growth. The experiments were repeated consecutively for two successive years to confirm the validity and reproducibility of the data.

#### 2.6.1 Measurement of biological parameters, grain attributes and total P content

The biological parameters including length and dry matter accumulation in roots and shoots of metal treated/untreated and inoculated/uninoculated wheat plants were measured. The lengths of plant organs and dry biomass were recorded at harvest. The total chlorophyll content in the fresh foliage of wheat was recorded at 90 days after sowing (DAS) according to the method described by ref. 33. The number of tillers, number of spikes, number of grains per spike, grain yield and straw yield were determined at harvest (110 DAS). Also, the protein content within the grains was estimated by the method of ref. 34. The phosphorus content in the dried roots and shoots of wheat plants was assessed at harvest by the vanadomolybdate method.^35^

#### 2.6.2 Estimation of proline, lipid peroxidation and antioxidant enzymes

The proline content in the fresh foliage of wheat was detected at 90 DAS following the method of ref. 36. The content of malondialdehyde (MDA) within the plant cells was determined by the extent of lipid peroxidation in plant tissues. Malondialdehyde was estimated according to the method of ref. 37. The absorbance of the orange-red supernatant was recorded at 532 nm, 600 nm and 450 nm. The molar extinction coefficient of MDA, *i.e.* 155 mM^−1^ cm^−1^, was employed for calculating MDA content and the result was expressed as μmol MDA g^−1^ fresh weight of tissue. Furthermore, antioxidant enzymes including superoxide dismutase (SOD), catalase (CAT) and glutathione reductase (GR) in the fresh foliage of wheat plants were quantitatively assayed at 90 DAS. The SOD activity was assayed by the method of ref. 38 and was expressed in terms of % increase in SOD activity over the control set. Catalase activity was determined by the method of ref. 39 where the decrease in the optical density of the plant extract mixed with H_2_O_2_ per minute was monitored at 240 nm and CAT activity was determined by using the molar extinction coefficient of H_2_O_2_ (36 mol^−1^ cm^−1^). Similarly, GR was estimated according to the method given by ref. 40. Glutathione reductase activity was reported in terms of oxidation of NADPH by monitoring the decrease in absorbance per minute at 340 nm. All enzyme assays were performed in triplicate.

#### 2.6.3 Heavy metal uptake

Accumulation of Ni and Pb in dried wheat organs (roots, shoots and grains) was measured at harvest.^41^ For this, 1 g samples of each dried plant tissue were digested separately in a mixture of nitric acid and perchloric acid (4 : 1). After complete digestion, the residual solution was filtered through Whatman no. 2 filter paper and the volume was made up to 100 mL with DDW and the metal content in each sample was determined by AAS employing CRM standards (JT Baker, BAKER INSTRA-ANALYZED Reagent Grade for Trace Metal Analysis) of Ni [1000 μg mL^−1^ (0.10% w/v)] and Pb [1000 μg mL^−1^ (0.10% w/v)].

### 2.7 Determination of factors/active biomolecules secreted by bacteria strain BM2 supporting wheat growth

#### 2.7.1 Production of indole acetic acid (IAA) and siderophores

The IAA secretion by metal tolerant bacterial strain BM2 was quantified by the modified method of ref. 42. For the assay, 100 μL of overnight grown culture was inoculated in 25 mL of Luria Bertani (LB) broth previously supplemented with 200 μg tryptophan mL^−1^ and treated with 0, 25, 50, 100, 200 and 400 μg mL^−1^ of Ni and Pb. Post-inoculation, the cultures were incubated for 48 h at 28 ± 2 °C in a shaking incubator at 125 rpm. Following incubation, 2 mL culture from each treatment was centrifuged at 10 000*g* for 15 min along with the addition of 2–3 drops of orthophosphoric acid and 4 mL of Salkowsky reagent (2% 0.5 M FeCl_3_ prepared in 35% HClO_4_) to the supernatant. Samples were kept in darkness for 1 h at 28 ± 2 °C for colour development. The IAA released in the supernatant was measured at *λ* = 530 nm by recording the absorbance of pink colour developed during the reaction using pure IAA as a standard. Furthermore, the siderophores were detected by the method of ref. 43 where chrome azurol S (CAS) agar plates amended with 0, 25, 50, 100, 200 and 400 μg mL^−1^ each of Ni and Pb were spot inoculated with 10^8^ cells per mL of strain BM2. After incubation, the plates were checked for yellow to orange zone (halo) formation around bacterial growth manifesting the release of siderophores. Also, a quantitative estimation of siderophores was done using Modi medium (K_2_HPO_4_ 0.05%; MgSO_4_ 0.04%; NaCl 0.01%; mannitol 1.0%; glutamine 0.1%; NH_4_NO_3_ 0.1%). For this, Modi medium enriched with 0, 25, 50, 100, 200 and 400 μg mL^−1^ of Ni and Pb was inoculated with 100 μL of bacterial cell suspension containing approximately 10^8^ cells per mL. The inoculated tubes were incubated for 5 days at 28 ± 2 °C. The cultures were then centrifuged at 6000 rpm and the catechol type phenolates [salicylic acid (SA) and 2,3-dihydroxybenzoic acid (DHBA)] in the supernatant were quantified.^44^

#### 2.7.2 Detection of hydrogen cyanide, ammonia and ACC deaminase

Hydrogen cyanide (HCN) secreted by strain BM2 was detected by the method of ref. 45. For this, a heavy inoculum was streaked on King's B agar plates containing 4.4 g L^−1^ glycine and amended with 0, 25, 50, 100, 200 and 400 μg mL^−1^ each of Ni and Pb. The lids of Petri plates were covered with filter paper discs dipped in 0.5% picric acid prepared in 2% sodium carbonate. Each experimental plate was sealed with parafilm so as to avoid the escape of volatile HCN released by the bacterial strain and incubated for 4 days at 28 ± 2 °C. The change in filter paper colour from yellow to orange was considered as a positive reaction. Similarly, for ammonia detection, the bacterial strain BM2 was inoculated in peptone water supplemented with 0, 25, 50, 100, 200 and 400 μg mL^−1^ each of Ni and Pb and incubated for 4 days at 28 ± 2 °C.^46^ The change in colour from yellow to reddish brown indicated a positive reaction.

The ACC deaminase produced by metal tolerant strain BM2 was qualitatively estimated by growing and spot inoculating bacterial culture on Dworkin and Foster (DF) salts minimal medium^47^ enriched with 3 mM ACC as the principal N source. The DF agar plates without ACC but containing (NH_4_)_2_SO_4_ (0.2% w/v) served as negative and positive control plates, respectively. Following incubation for 72 h at 28 ± 2 °C, the plates were monitored for bacterial growth. *Mesorhizobium* LMS-1 containing pRKACC plasmid^48^ was employed as a positive control. Quantitative estimation of ACC deaminase was done by the modified method of ref. 49. The concentration of α-ketobutyrate released due to breakdown of ACC was measured by comparing the absorbance of samples against a standard curve of pure α-ketobutyrate. The ACC deaminase activity was expressed as μmol α-ketobutyrate mg^−1^ protein h^−1^.

### 2.8 Statistical analysis

Each *in vitro* experiment was repeated three times and the data was statistically analyzed by one way ANOVA (*P* ≤ 0.05) using Minitab 17 statistical software. Also, the wheat data was subjected to Duncan's multiple range test (DMRT) at 5% probability level to compare the treatment means.

## 3. Results and discussion

### 3.1 Heavy metal concentration and microbial diversity of experimental soil

The nickel and lead concentration in contaminated soils collected from the industrial area (S1) was 34.9 and 16.0 μg g^−1^, respectively, whereas it was significantly (*P* ≤ 0.05) lower at 16.7 and Pb 9.5 μg g^−1^ in conventional soil (S2). The microbial composition differed significantly among S1 and S2 (*P* ≤ 0.05) and included nitrogen fixers, phosphate solubilizers, soil rhizobia, other bacteria and fungal populations. Comparatively, the soil microbial composition was poorer in S1 than S2, probably due to the high levels of heavy metals present in S1, which might have reduced the overall microbial load (Table 1). Comparing the microbial variations in both the rhizospheric soils, S1 had the highest number of actinomycetal populations (1100 × 10^4^) whereas rhizobia dominated in the S2 rhizosphere (Table 1) as compared to other groups of microbiota. Overall, a significant physiological difference between the microbial populations in conventional and metal polluted soils was recorded, which could possibly be due to the differences in nutrient uptake ability, preference for macro and micro nutrient elements of soils and metabolic activities of the microbes inhabiting S1 and S2 rhizospheres. Many other studies have also found similar variations in soil microbial community structures populating polluted and non-polluted soils.^50,51^

**Table 1:** Heavy metal concentrations and microbial compositions of metal polluted and conventional soils^a^

Microbial composition (cfu g^−1^ soil)										
			*Pseudomonas* (×10^5^)	*Rhizobium* (×10^5^)	*Bacillus* (×10^5^)	*Azotobacter* (×10^5^)	Fungi (×10^4^)	Actinomycetes (×10^4^)	Phosphate solubilizers (×10^4^)	
									PSB	PSF
S1 rhizosphere			700	100	600	200	200	1100	800	100
*Pennisetum glaucum*										
S2 rhizosphere			2000	3500	2300	2200	200	700	1100	200
Conventional soil										
Heavy metals (μg g^−1^)										
	Ni	Pb								
S1	34.9	16.0								
S2	16.7	9.5								

a Values are mean of three independent replicates.

### 3.2 Heavy metal tolerance and bacterial strain identification

A total of 10 bacterial strains isolated in the present study displayed a variable metal tolerance profile. Among all cultures, strain BM2 demonstrated maximum tolerance and could survive even at the highest concentrations of Ni (1600 μg mL^−1^) and Pb (2000 μg mL^−1^). The ability of this strain to survive at maximum metal levels is interesting because such an organism could be used as an inoculant for enhancing the production of crops even under metal stress. In similar studies, *Gemella* sp., *Micrococcus* sp., and *Hafnia* sp. have been found to exhibit a variable pattern of tolerance towards Cd, Cr and Pb.^52^ Likewise, *B. cereus* and *B. amyloliquefaciens* could tolerate very high concentrations (up to 2000 μg mL^−1^) of Pb whereas *P. aeruginosa*, *Chryseobacterium* sp., and *B. subtilis* tolerated 1200 μg mL^−1^ each of Cd and Ni.^53^ The reason why microbial populations differ so hugely in their ability to tolerate such deadly pollutants is a topic of global debate and research. It has been suggested that such variable metal tolerance behaviour occurring naturally among microbes towards various heavy metals could be due to (i) the differences in available macro and micro nutrient elements in growth media/soils supporting bacterial growth (ii), variable genetic constituents of bacterial strains and (iii) the growth conditions/environmental variables affecting the bacterial growth.^54^ In addition to these, the metal tolerant microbes, which could serve as a bioremediation tool, are capable of complexing metals inside the cell, pumping out the metal ions to the exterior of the cell and enzymatically degrading/transforming certain metal ions. These are some of the strategies, acting independently or simultaneously, by which metal tolerant bacteria can obviate the detrimental impact of metals on soil and plant health, thereby minimizing human health hazards.^55^ The metal tolerant strain BM2 was subsequently characterized and molecularly identified to species level. Strain BM2 was a Gram positive and rod shaped bacterium which produced rough and dry colonies with wavy margins on nutrient agar plates (Table 2). The morphological (shape, colour and morphology of bacterial colonies) and biochemical (indole, Voges–Proskauer, nitrate reduction, starch and lipid hydrolysis *etc.*) properties as displayed in Table 2 confirmed strain BM2 as *Bacillus* sp. while it was identified to species level as *Bacillus subtilis* (GenBank accession number MF589974) by 16SrRNA gene sequence analysis.

**Table 2:** Morphological, biochemical and molecular characterization of metal tolerant strain BM2^a^

**Morphological characteristics**	
Colony shape	Irregular
Colony colour	Off white
Colony morphology	Wrinkled with wavy margins
Pigmentation	None
Gram reaction	Gram positive
Cell shape	Long rods

**Biochemical characteristics**	
Indole	−
Methyl red	−
Voges–Proskauer	+
Citrate utilization	+
Nitrate reduction	+
Catalase	+
Oxidase	−
Urease	−
Starch hydrolysis	+
Lipid hydrolysis	+

**Molecular characterization**	
Molecularly identified as: *B. subtilis* (GenBank accession number MF589974)	

a ‘+’ and ‘−’ indicate positive and negative reactions respectively.

### 3.3 Cellular damage and metal localization within strain BM2

**Fig. 1:**
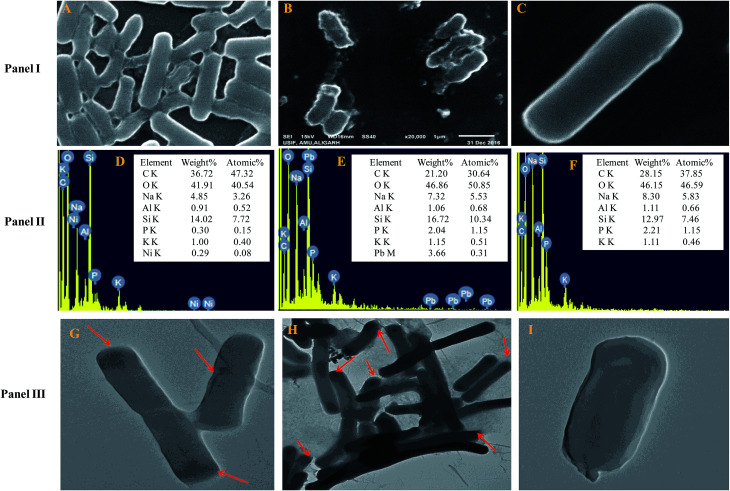
Panel I shows cell surface morphology alterations revealed by SEM micrographs of *B. subtilis* BM2 cells treated with 200 μg mL^−1^ Ni (A) and 400 μg mL^−1^ Pb (B) and an untreated control (C). Panel II displays the EDX spectra and the percentage of various elements in Ni treated (D), Pb treated (E) and untreated cells (F) of *B. subtilis* BM2. Panel III represents TEM micrographs of *B. subtilis* BM2 treated with 200 μg mL^−1^ Ni (G), 400 μg mL^−1^ Pb (H) and an untreated control (I). The darker regions marked with red arrows indicate possible areas of metal distribution and localization within treated cells.

The cells of *B. subtilis* BM2 exhibited disrupted cellular morphology when grown in NB amended with 200 μg mL^−1^ Ni (Fig. 1 Panel I A) and 400 μg mL^−1^ Pb (Fig. 1 Panel I B) as compared to the untreated control cells (Fig. 1 Panel I C) when viewed under SEM. The metal loaded cells of *B. subtilis* displayed shrinkage and had elongated membranes along with other surface distortions relative to the unloaded (control) bacterial cells, whose membrane was smooth and without any deformity. In a similar study, morphological disruptions in the cells of *Pseudomonas stutzeri* MTCC101 were revealed under SEM when cells were exposed to higher doses of Cd.^56^ Additionally, reduction in bacterial cell size when cultured in the presence of Cd and Ni^57^ and Pb^58^ has been reported. The cell elongation/shrinkage in bacterial cells following exposure to metal ions could possibly be an adaptive strategy employed by bacteria to safeguard themselves from the toxic impact of metals in the environment. This feature is unusual in that, despite the damage and disruptive effects of heavy metals, the cells of *B. subtilis* BM2 could localize some percentage of metals inside their tissues, which may be a metal removal strategy adopted by this bacterium that could be employed to remediate metal contaminated environments. The EDX spectra showing the accumulation of Ni and Pb within the cells are presented in Fig. 1 Panel II D and Panel II E, respectively, compared to the control (Fig. 1 Panel II F).

Transmission electron microscopy is yet another powerful technique which provides a 1000-fold improved resolution of the image and thus allows a clear visualization of the finer details of bacterial cells. Historically, TEM with its very high resolution efficiency has been found to serve as a precise technique for understanding the cell architecture and localization of metals within cells.^59^ Considering this, the toxicity of Ni and Pb towards *B. subtilis* BM2 was further validated by TEM imaging of the cells grown in the presence of 200 μg mL^−1^ Ni (Fig. 1 Panel III G) and 400 μg mL^−1^ Pb (Fig. 1 Panel III H) and compared with untreated cells (Fig. 1 Panel III I). In the images, the darker regions confirmed the distribution of metals in that particular region. Similar to the results obtained in this study, surface distortions and deposition of metals inside *Enterobacter* sp. strain EG16 cells while growing in the presence of Cd have previously been shown under TEM.^60^ Also, the presence of Pb within the cells of *Klebsiella* sp. 3S1 following exposure to Pb has been revealed under HR-TEM.^61^

**Fig. 2:**
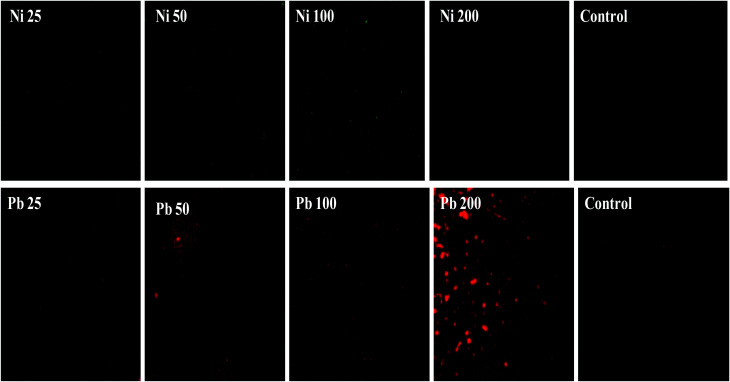
Cellular death of *B. subtilis* BM2 cells grown with various concentrations of Ni and Pb and stained with acridine orange and propidium iodide and compared with an untreated control.

Furthermore, the metal toxicity to *B. subtilis* BM2 was also expressed in terms of increasing number of dead cells with progressively increasing metal concentrations (Fig. 2). The image shows the increase in red fluorescence of PI stained dead cells. Since PI (a DNA intercalating dye) can only permeate through membrane compromised cells, the healthy untreated cells did not show any red fluorescence of PI. For this reason, PI is an excellent indicator dye to detect metal toxicity. A similar increase in the number of dead cells of *Burkholderia cepacia* strain PSBB1 with progressively increasing concentrations of glyphosate has been reported.^62^ From the current experiments it was, however, clear that despite the damage to *B. subtilis* BM2 cells inflicted by Ni and Pb, observed under several accurate and powerful techniques such as SEM, TEM and CLSM, the bacterial strain still survived and displayed considerable potential for metal removal from the metal stressed environments. Hence, this intrinsic property of these bacterial cells could be exploited to remediate contaminated environments.

### 3.4 Metallothionein induction by *B. subtilis* BM2 as a metal detoxification mechanism

MTs, a class of low molecular weight, cysteine rich proteins (generally ranging from 3500–14 000 Da) consisting of approximately 61–68 amino acids, have been found distributed abundantly among living organisms.^63^ Mechanistically, the thiol groups of MTs bind to heavy metal ions, and thus make heavy metals unavailable to microbes.^64^ By doing this, MTs shield the bacterial cells from the lethal impact of metals and oxidative stress, which could otherwise pose a disastrous risk to their survival. Considering the importance of MTs in metal detoxification, *B. subtilis* strain BM2 was screened to see whether this strain was able to induce MTs production while growing in the presence of Ni (200 μg mL^−1^) and Pb (400 μg mL^−1^). Indeed, *B. subtilis* BM2 exhibited greater production of MTs in the presence of both Ni and Pb, suggesting an obvious role of metals in MTs induction (Table 3). In contrast, MTs were secreted in lower amounts by untreated/control cells (0.4 μmol) compared to those released by *B. subtilis* BM2 when grown in the presence of Ni (3.1 μmol) and Pb (3.6 μmol) (Table 3). Of the two metals, Pb was however, found, to be a superior inducer of MTs compared to Ni. It is clear that the secretion of MTs is a crucial mechanism adopted by microbes to remediate metal contaminated sites while surviving in metal polluted environments. In agreement with our findings, the study in^31^ reported a similar induction of MTs by *B. cereus* cells cultured in the presence of varying doses of Pb. Similar induction of MTs in *P. aeruginosa* and *P. putida* cells upon exposure to Cu and Cd has been reported by ref. 64. The results obtained in this study and reported elsewhere by others, therefore, clearly establish the fact that MTs so generated by bacterial communities in stressed environments could be useful in efficiently detoxifying metal-contaminated environments.^65^ Hence, the use of bacteria possessing this property of secreting MTs could be explored as a cost effective approach in bioremediation strategies.

**Table 3:** Estimation of metallothionein (MT) content in metal loaded and unloaded cells of *B. subtilis* BM2^a^

Treatment	*x*-value from curve equation	MT content (μ mol) [*x*-value/20]
Control	8.3^c^ ± 0.6	0.4^c^ ± 0.03
Ni 200 μg mL^−1^	62.8^b^ ± 1.7	3.1^b^ ± 0.09
Pb 400 μg mL^−1^	71.1^a^ ± 2.6	3.6^a^ ± 0.10
LSD	2.8	0.14
*F*-value (treatment)	2408.9	2408.9

a Values are mean of three independent replicates; ± indicates standard deviation. Values denoted with different letters are significantly different at *P* ≤ 0.05.

### 3.5 Cellular damage, metal localization and morphology alterations of plant organs observed under SEM, EDX and CLSM

Morphological damage to the leaf and root tissues of wheat plants was observed when the plants were grown on soft agar plates supplemented with 100 μg mL^−1^ Ni and 200 μg mL^−1^ Pb and visualized under SEM. Similar results depicting structural distortions in the roots of wheat plants grown under Pb stress have previously been reported.^66^

**Fig. 3:**
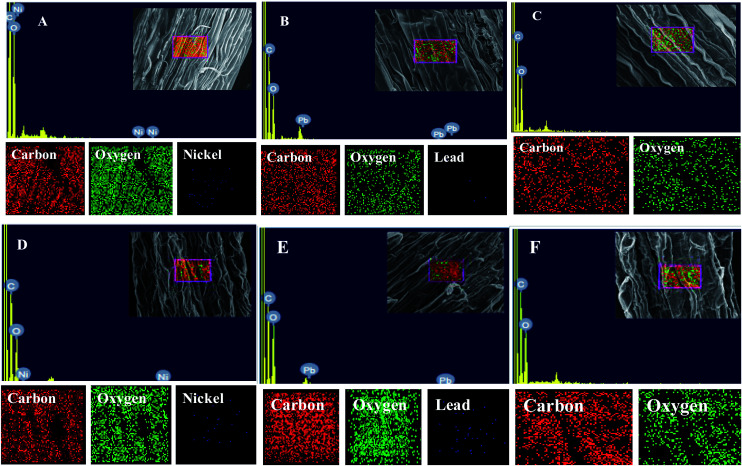
EDX and elemental analysis of leaf (A–C) and root (D–F) tissues of wheat. (A) and (B) represent mapping and EDX spectra of leaf tissue treated with 100 μg mL^−1^ Ni and 200 μg mL^−1^ Pb, respectively. (D) and (E) represent mapping and EDX spectra of root tissue treated with 100 μg mL^−1^ Ni and 200 μg mL^−1^ Pb, respectively. (C) and (F) represent mapping and EDX spectra of untreated leaf and root tissues of wheat, respectively.

**Fig. 4:**
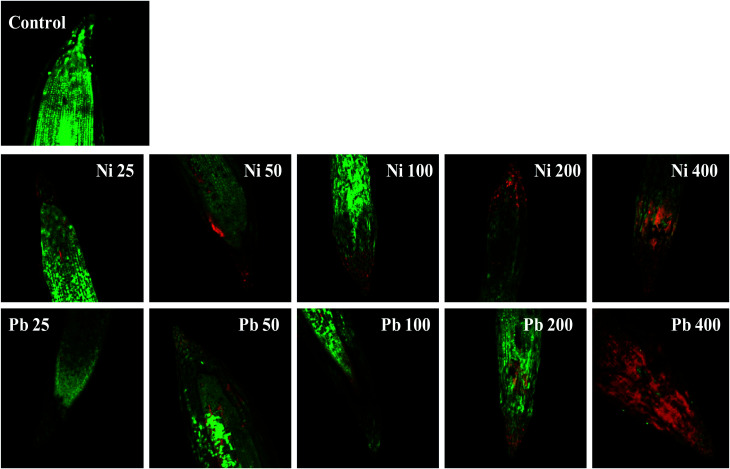
Oxidative stress and cell death in wheat roots stained with propidium iodide (PI) and acridine orange (AO) and treated with 25–400 μg mL^−1^ each of Ni and Pb and compared with an untreated control. The image shows the number of dead cells, which increased with an increase in metal concentration, as revealed by red fluorescence of PI when observed under CLSM.

Energy dispersive X-ray spectroscopy (EDX) is another powerful tool which has widely been employed to unravel the finer details of living tissues including the toxicity caused by stressor molecules like heavy metals.^67,68^ Moreover, EDX coupled to either TEM or SEM provides useful information about the chemical formulations/elemental compositions of organisms. Due to this distinctive feature, EDX in a recent study was used to determine the nutrient utilization and distribution of elements (including heavy metal ions) in biological samples.^69^ Considering the efficacy of EDX in various microscopic examinations, the distribution and location of metals in the leaf and root tissues of wheat grown *in vitro* was assessed by this method. The elemental analysis of the leaf and root tissues of wheat was done following heavy metal exposure (Fig. 3) and the results showed that lesser amounts of metals could be accommodated within the leaf tissues as compared to the root tissues. Conversely, a higher level of damage was detected in roots compared to leaves since roots are the primary organs to come in direct contact with the metal ions present in the surrounding growth medium. However, the region of damage caused due to metal toxicity differed between the two plant organs. Similar results have also been reported by ref. 70 who described the localization of metals, especially Pb, within and around the root, petiole and leaf surface of *Eichhornia crassipes* when observed through SEM-EDX. In another study, the elemental composition of the roots, stems and leaves of *Corchorus tridens* plants were detected using energy dispersive X-ray fluorescence (EDXRF).^71^ In the present study, the roots of Ni and Pb treated wheat plants also exhibited oxidative stress resulting in tissue death as revealed by CLSM. Interestingly, a concentration dependent increase in the number of dead cells of wheat root tissues (indicated by the red fluorescence of PI, which was taken up only by the dead cells) was observed under CLSM (Fig. 4) when the plants were exposed to Ni and Pb stress. PI can serve as an important tool to differentiate between live and dead tissues as it cannot cross the membrane of live cells, and thus displays a clear picture of oxidative damage caused by heavy metals or any other stressor agents. Also, the roots of the untreated control plants exhibited maximum intensity of green fluorescence of acridine orange (Fig. 4), indicating very little or almost nil cellular damage in the untreated control. In accordance with our findings, oxidative stress in roots detached from other plants, including cereals, while growing in the presence of various stressors has recently been reported.^72–74^

### 3.6 Metal toxicity and bioremediation studies on wheat

#### 3.6.1 Biological parameters, photosynthetic pigments and total P content

**Fig. 5:**
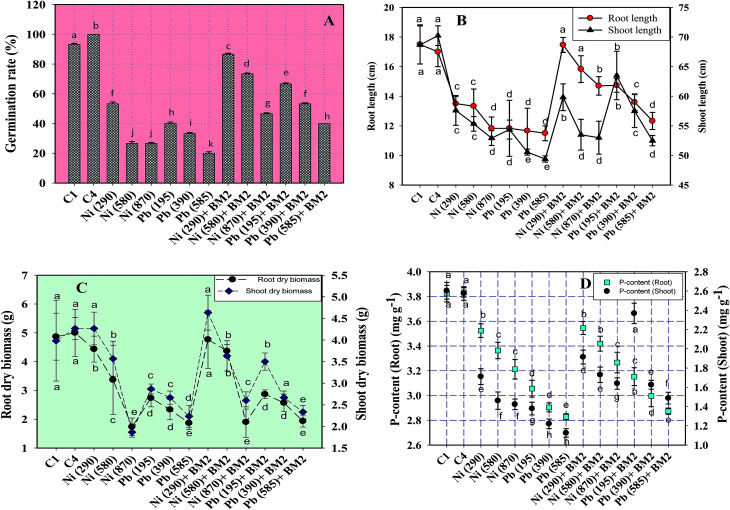
Toxic impact of three doses (mg kg^−1^ soil) of Ni and Pb (dose rates mentioned within brackets) on the biological parameters of wheat plants and bioremediation by metal tolerant *B. subtilis* BM2: germination percentage (A); length of roots and shoots (B); dry matter accumulation in roots and shoots (C); total P content within roots and shoots (D). C1 and C4 represent uninoculated and *Bacillus* inoculated controls, respectively. Values denoted with different letters are significantly (*P* ≤ 0.05) different according to Duncan's multiple range test.

The impact of exposure to Ni and Pb on the wheat plants was variable. Ni at 870 and Pb at 585 mg kg^−1^ inhibited the germination of wheat seedlings by 71.4% and 78.6%, respectively, with respect to the uninoculated control. However, *B. subtilis* BM2 used as a bioinoculant enhanced the germination rate by 42.8% and 50%, respectively, at the same doses of Ni and Pb (Fig. 5A). Likewise, the root length of metal treated wheat plants declined by 32.6% and 34.3% at 870 mg Ni and 585 mg Pb kg^−1^, respectively, when compared with the uninoculated control, which, however, improved by 19.7% and 6.5% following inoculation with *B. subtilis* BM2 (Fig. 5B). Moreover, Ni at 870 mg kg^−1^ and Pb at 585 mg kg^−1^ reduced the shoot length of wheat plants by 23% and 28%, respectively, when compared with uninoculated and untreated control plants. The *B. subtilis* inoculated plants however had better shoot growth (Fig. 5B). Similarly, Ni at 290 mg kg^−1^ reduced the dry matter accumulation in the roots and shoots of wheat plants by 10.2% and 30.8% respectively over the control. The dry biomass of *B. subtilis* inoculated roots and shoots was enhanced by 8.3% and 6.5% even at 290 mg Ni kg^−1^ (Fig. 5C).

Similarly, the dry matter accumulation in the roots and shoots decreased by 44.9% and 25.6%, respectively, when wheat plants were grown in the presence of 195 mg Pb kg^−1^ as compared to the uninoculated control. Interestingly, when *B. subtilis* BM2 was added to the metal treated experimental soils, the root and shoot dry biomass enhanced by 6.9% and 17.1% respectively (Fig. 5C). In agreement with our findings, an earlier study^75^ reported a similar decline in various biological parameters of wheat plants grown in the presence of exogenously applied chromium. Moreover, in yet another study, a concentration dependent decline in germination rate, length of plant organs, fresh and dry biomass and total protein content of maize plants grown in the presence of increasing concentrations of Pb has been reported.^76^ Also,^77^ reported a significant enhancement in the growth parameters, including the rate of germination, in wheat seedlings exposed to Cd stress following inoculation with *Pseudomonas* sp. These findings suggest that the inoculation of the culture with *B. subtilis* BM2 diminished/eliminated the heavy metal toxicity due to its metal detoxifying ability and, thus, improved various parameters of wheat plants grown in metal treated soils.

Moreover, the total chlorophyll content of the fresh foliage of wheat plants grown under Ni and Pb stress decreased significantly (*P* ≤ 0.05) by 15.6% and 37.5% in the presence of 290 mg Ni kg^−1^ and 195 mg Pb kg^−1^ soil, respectively, compared to the uninoculated control (Fig. 7A). However, when compared with the uninoculated plants, the foliage of inoculated plants displayed greater amounts of chlorophyll. The chlorophyll content in plants inoculated with *B. subtilis* BM2 increased by 7% even in the presence of 290 mg Ni kg^−1^. Likewise, strain BM2 inoculation resulted in successful bioremediation of metal treated experimental soil and improved the chlorophyll content by 13% even at 195 mg Pb kg^−1^ soil (Fig. 7A). A similar study demonstrating the disturbance in photosynthetic activity of durum wheat cultivated in the presence of Cd and Zn stress has been reported.^78^ In contrast, the study in ref. 79 found a substantial increase in leaf photosynthetic pigments and other vital growth parameters of *P. aeruginosa* inoculated wheat grown under Zn stress. In another similar study, improved chlorophyll content in the foliage of *Zea mays* following PGPR inoculation even in the presence of various metals has also been reported.^80^ Herein, when investigating the impact of Ni and Pb on the total P content in the organs of wheat plants, it was observed that the P content in roots declined by 15.8% when the plants were grown with 870 mg Ni kg^−1^ and by 26.3% in the presence of 585 mg Pb kg^−1^. In contrast, inoculation with *B. subtilis* BM2 improved the P content marginally within the roots by 3% and 3.4% with 870 mg Ni kg^−1^ and 585 mg Pb kg^−1^ respectively (Fig. 5D). The shoots of wheat plants accumulated lower quantities of P as compared to the roots since roots are the primary organs that, along with water, directly absorb other nutrients present within the soil-plant continuum. The total P content in shoots declined by 46.2% in the presence of 870 mg Ni kg^−1^ and by 57.7% at 585 mg Pb kg^−1^ when compared with the uninoculated control. Interestingly, *B. subtilis* BM2 improved the P content in shoots by 12.5% and 26.7% even at 870 mg Ni kg^−1^ and 585 mg Pb kg^−1^, respectively (Fig. 5D). In a similar study, a considerable increase in total P content of roots and shoots of maize plants due to inoculation with metal tolerant *A. chroococcum* CAZ3 under Cu and Pb stress has been reported.^74^

#### 3.6.2 Number of tillers, spikes, straw and grain attributes

**Fig. 6:**
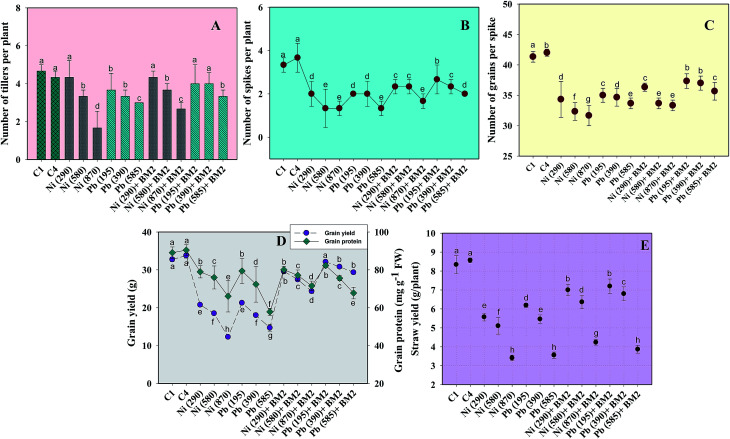
Effect of three doses (mg kg^−1^ soil) of Ni and Pb (dose rates mentioned within brackets) on various parameters of wheat plants and bioremediation by metal tolerant *B. subtilis* BM2: number of tillers per plant (A); number of spikes per plant (B); number of grains per spike (C); grain yield and grain protein (D); straw yield (E). C1 and C4 represent uninoculated and *Bacillus* inoculated controls, respectively. Values denoted with different letters are significantly (*P* ≤ 0.05) different according to Duncan's multiple range test.

Generally, a dose dependent decrease in the number of tillers and spikes was observed for wheat plants while growing with varying doses of Ni and Pb. Nickel at 870 mg Ni kg^−1^ maximally reduced the number of tillers by 64% as compared to the uninoculated control. In contrast, *B. subtilis* BM2 applied as a bioinoculant could remediate the contaminated soils and consequently increase the number of tillers by 37% even in the presence of 870 mg Ni kg^−1^ (Fig. 6A). The number of tillers in plants grown at 290 mg kg^−1^ Ni, however, did not increase even after *B. subtilis* inoculation. Likewise, *B. subtilis* inoculation caused a considerable enhancement of 17.5% in the number of tillers when plants were grown with 390 mg Pb kg^−1^ soil (Fig. 6A). Like for vegetative growth, heavy metals also exerted a negative impact on the reproductive parameters of wheat plants including the spikes, grains per spike and grain yield. A significant reduction of 60.6% in the number of spikes was recorded when wheat plants were grown with 870 mg Ni kg^−1^ compared to the control. In contrast, *B. subtilis* enhanced the number of spikes by 23.5% (Fig. 6B). Similarly, in the presence of 585 mg Pb kg^−1^, the number of spikes was reduced by 60.6% when compared with the uninoculated control while *B. subtilis* inoculation showed 35% improvement at the same dose of Pb (Fig. 6B). Moreover, metal tolerant bioinoculant *B. subtilis* exhibited an increase of 19% and 10.3% in the straw yield over uninoculated plants even in the presence of 870 mg Ni kg^−1^ and 585 mg Pb kg^−1^, respectively (Fig. 6E). Furthermore, the number of grains per spike also reduced in the presence of heavy metals. The maximum reduction in number of grains, grain yield and grain protein content was exhibited by nickel: specifically, these declined by 23%, 62% and 26% at 870 mg Ni kg^−1^, respectively, relative to the uninoculated control. In contrast, *B. subtilis* inoculation improved the number of grains, grain yield and grain protein content by 5%, 49% and 8%, respectively (Fig. 6C and D). Protein synthesis is inhibited under high doses of heavy metals, most likely due to binding of metals to the sulphydryl groups of proteins, wherein the normal structure of the protein is destroyed. This is probably why the protein content declined consistently with a progressive increase in the dose of metals applied to the experimental soils. A similar adverse impact of As on various biological and chemical properties of wheat has been reported by ref. 81. In contrast, alleviation of heavy metal stress and growth promotion of wheat by *Bacillus* sp. strain USTB-O grown under the influence of Cu stress has also been reported.^82^

#### 3.6.3 Proline content and lipid peroxidation in metal stressed wheat foliage

**Fig. 7:**
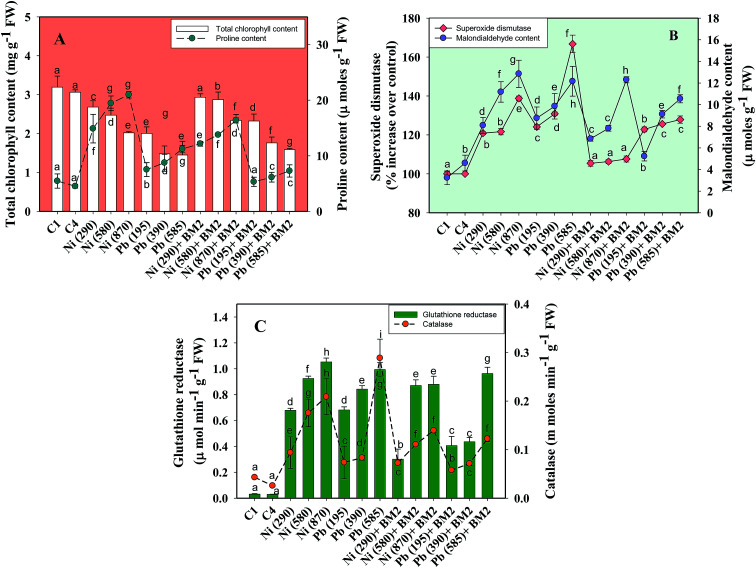
Lethal impact of three doses (mg kg^−1^ soil) of Ni and Pb (dose rates mentioned within brackets) on chlorophyll, proline and antioxidant defence response detected in the foliage of wheat plants and bioremediation by metal tolerant *B. subtilis* BM2: total chlorophyll and proline content (A); MDA and SOD activity (B); GR and CAT activity (C). C1 and C4 represent uninoculated and *Bacillus* inoculated controls, respectively. Values denoted with different letters are significantly (*P* ≤ 0.05) different according to Duncan's multiple range test.

Proline is reported to serve as an osmoprotectant which guards plants from the deleterious effects of ROS generated by plants under the influence of environmental stresses like heavy metals, drought, salinity *etc.*^83^ Also, proline assists plants in maintaining osmotic balance and homeostasis.^84^ Besides these, proline acts as an ROS scavenger, and plays a crucial role in stabilizing sub-cellular structures of plants surviving under various environmental stresses.^85^ Considering these, the proline content in the fresh foliage of wheat plants grown with/without Ni and Pb was quantified. Generally, the proline content in wheat foliage increased progressively with an increase in heavy metal stress. Interestingly, a 74% and 52% increase in proline content in fresh foliage was recorded when wheat plants were grown in the presence of 870 mg Ni kg^−1^ and 585 mg Pb kg^−1^, respectively, relative to the uninoculated control (Fig. 7A). In a similar study, accumulation of elevated levels of proline in the leaves of wheat plants grown under drought stress was reported.^86^ However, after application of *B. subtilis* BM2, the proline levels were reduced substantially by 22% and 35% even when the wheat was grown with 870 mg Ni kg^−1^ and 585 mg Pb kg^−1^, respectively, relative to the uninoculated plants. In agreement with our results, a similar reduction in proline content in the shoots of wheat plants inoculated with *A. chroococcum* and grown under stressed environments was reported.^87^ The metal tolerant *B. subtilis* used in this study significantly lowered the levels of proline in metal stressed wheat plants as the bacterial strain ameliorated the heavy metal stress and protected the plants from oxidative damage, allowing them to grow even under metal stress. These results underpin the fact that strain BM2 could act as a potential organism in bioremediation strategies, leading eventually to the overall improvement in the performance of wheat cultivated under metal stress.

Heavy metal stress triggers ROS generation in many plants including wheat, which influences lipid peroxidation within the cell membrane. Malondialdehyde (MDA) is one such lipid peroxidation product in plant tissues. Therefore, MDA also acts as an indicator of stress severity for plants.^88^ Taking this into consideration, the formation of MDA in leaves collected from wheat plants grown in soils treated with different doses of Ni and Pb was assayed. In general, the MDA content in leaves increased with increasing dose of metals. For instance, Ni at 870 mg kg^−1^ increased the MDA content profoundly by 75% compared to the uninoculated control (Fig. 7B) while Pb at 585 mg kg^−1^ enhanced the MDA content by 74% over the control. However, *B. subtilis* BM2, when applied, alleviated the metal stress and concomitantly lowered the MDA levels by 4.7% even in the presence of 870 mg Ni kg^−1^, while at 585 mg Pb kg^−1^ it reduced the MDA content by 13.9% in inoculated plants relative to uninoculated plants (Fig. 7B). In analogous experiments, a significant enhancement in the MDA content of wheat plants grown under Al^89^ and Zn stress^79^ has been reported.

#### 3.6.4 Antioxidant enzyme activity

Generally, a dose dependent increase in the antioxidant enzyme activity of foliage was recorded when inoculated or uninoculated wheat plants were cultivated in the presence of varying concentrations of Ni and Pb. Comparing the two metals, the maximum SOD was found in the presence of Pb whereas the GR and CAT activity were higher in Ni treated plants, suggesting the severity of stress faced by wheat plants grown under varying doses of Ni and Pb. Ni induced a CAT value of 0.16 mmol min^−1^ mg^−1^ protein and GR activity of 0.9 μmol min^−1^ g^−1^ FW (Fig. 7C). Interestingly, in the presence of *B. subtilis* BM2 the metal stress was relieved, and hence, the accumulation of antioxidant enzymes in metal treated wheat plants was greatly reduced. For example, the GR activity of *B. subtilis* BM2 inoculated plants was diminished maximally by 14% at 870 mg Ni kg^−1^ compared to the uninoculated plants (Fig. 7C). The SOD activity was increased by 27% at three times the concentration of Ni compared to the uninoculated control (Fig. 7B). Interestingly, following *B. subtilis* inoculation, the reduction in SOD activity of wheat foliage was 80% at 870 mg Ni kg^−1^ and 58% at 585 mg Pb kg^−1^ (Fig. 7B). In conclusion, it can very safely be argued that the bacterial inoculant *B. subtilis* BM2 alleviated the heavy metal stress and, therefore, reduced the level of antioxidant enzymes, which otherwise were augmented under metal stress situations. The obvious decline in antioxidants due to inoculation with metal tolerant *B. subtilis* strain BM2 consequently resulted in a sizeable increase in overall growth of the wheat plants even in metal contaminated soils. The similar beneficial role of *Bacillus subtilis* SU47 and *Arthrobacter* sp. SU18 in reducing the antioxidant enzyme activity in wheat plants by alleviating stress^90^ validates our findings.

#### 3.6.5 Heavy metal uptake

**Fig. 8:**
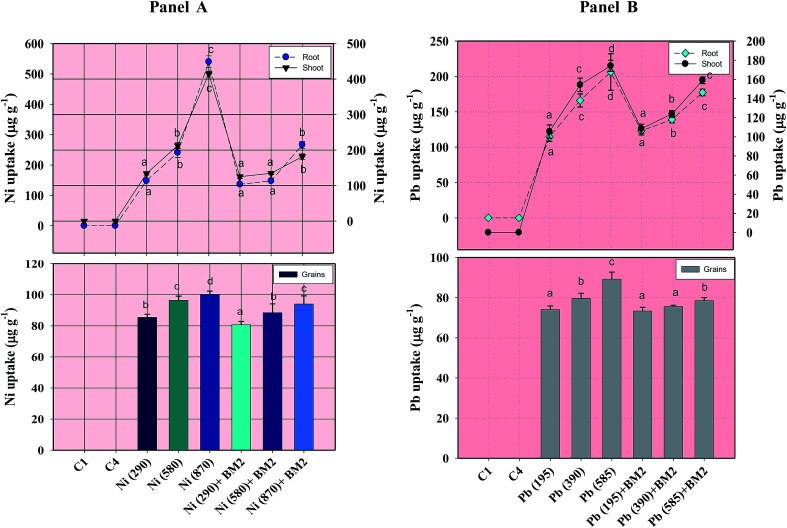
Concentration of Ni (Panel A) and Pb (Panel B) within roots, shoots and grains of wheat plants grown with and without inoculum and detected at harvest. C1 and C4 represent uninoculated and *Bacillus* inoculated controls, respectively. Values denoted with different letters are significantly (*P* ≤ 0.05) different according to Duncan's multiple range test.

The concentration of metals within various organs of the wheat plants differed considerably but, however, increased consistently with increasing doses of Ni (Fig. 8 Panel A) and Pb (Fig. 8 Panel B). Among plant organs, roots in general had the highest concentration of Ni (309.6 μg g^−1^) followed by shoots (253.7 μg g^−1^) and grains (94.0 μg g^−1^). A trend similar to Ni was also recorded for Pb. Interestingly, the grains of both inoculated and uninoculated plants contained the least amounts of both metals. In contrast, inoculation with *B. subtilis* BM2 reduced, by 51%, the metal uptake by roots detached from the plants grown at 870 mg Ni kg^−1^. Similarly, in the presence of 585 mg Pb kg^−1^, a 14% drop in the metal deposition within the roots of inoculated plants was observed. Although Pb is a highly toxic metal, Pb ions are easily transported from soil to various plant parts. This could possibly be due to the release of protons, root exudates and other metabolites, which results in easy dissolution of Pb ions, probably through the formation of metal chelating complexes.^91^ The organ-wise accumulation of heavy metals followed the order: roots > shoots > grains. Similar accumulations of other metals like Zn, Cr, Fe, Ni and Cd within various organs of other cereal crops like maize,^92^ rice^93^ and wheat^94^ have been reported. Herein, the same trend of metal accumulation within various organs of wheat was observed even after inoculation with *B. subtilis* BM2. Interestingly, following inoculation, the grains displayed the maximum reduction in metal accumulation and a 6.2% decrease in grain Ni concentration was recorded when inoculated plants were grown in the presence of 870 mg Ni kg^−1^. Likewise, a 12% decline in Pb content within grains was observed when the wheat was raised in 585 mg Pb kg^−1^-containing soil, relative to the uninoculated plants (Fig. 8). Generally, *B. subtilis* BM2 inoculated plants displayed lesser deposition of metals in each organ, possibly because the metal tolerant strain might have accumulated most of the metal fraction present within the soil. As a result, a lower quantity of metal was available for uptake by the plants. In this way, when metal pressure was relieved, the inoculated wheat plants could survive better even in metal treated soils.

### 3.7 Assessment of factors involved in wheat growth promotion under metal stress

#### 3.7.1 Indole acetic acid and siderophores

Metal tolerant strain *B. subtilis* BM2 could synthesize variable amounts of growth promoting biomolecules, which supported the growth of wheat plants grown under heavy metal pressure (Table 4). Among various plant growth enhancers, IAA was maximally produced by strain BM2 when cultured in metal free medium (51.6 μg mL^−1^), which, however, declined by 43.6% and 49% in the presence of 100 μg mL^−1^ each of Ni and Pb, respectively (Table 4). Comparing the impact of varying concentrations of metals on IAA secretion by strain BM2, the highest tested concentration of the two metals had the most lethal effect on IAA production. Specifically, Pb at 400 μg mL^−1^ exhibited the most inhibitory impact and reduced IAA by 58% while Ni at the same concentration reduced the IAA production by 47% when compared with the metal free control (Table 4). It was, therefore, evident from the present findings that despite the toxicity of these metals, the truncated strain BM2 maintained the plants' secretion of IAA into the exterior environment even while growing at higher concentrations of Ni and Pb. However, the amount of IAA diffused exteriorly was poorer under metal stress than under normal metal free conditions. Like strain BM2, many bacteria colonizing the rhizospheres have been found to produce IAA^95^ and positively affect many vital physiological processes of plants.^96^ Hence, the IAA-promoting ability of this metal tolerant strain *B. subtilis* BM2, in addition to its capability to relieve heavy metal stress, could be an interesting option for enhancing the growth of wheat plants even under metal stressed situations. The IAA released by soil microbiota has been reported to influence plant growth by accelerating the division and enlargement of cells, and inducing root elongation and apical dominance in wheat plants, even when grown in metal polluted soils.

**Table 4:** Assessment of active biomolecules secreted by *B. subtilis* strain BM2 under metal pressure^a^

Heavy metal	Dose rate (μg mL^−1^)	IAA* (μg mL^−1^)	Siderophore			ACC deaminase (μM α-ketobutyrate mg^−1^ protein h^−1^)	HCN	NH_3_
			CAS agar (mm)	Phenolates (μg mL^−1^)				
				SA	2,3-DHBA			
Control	00	51.6^a^ ± 0.9	11.3 ± 0.6	54.8^a^ ± 3.4	3.6^a^ ± 0.4	209.5^a^ ± 1.0	+	++
	25	37.1^b^ ± 0.6	ND	41.6^b^ ± 1.1	3.2^b^ ± 0.1	134.8^b^ ± 3.9	ND	++
	50	32.6^c^ ± 1.1	ND	39.2^b^ ± 0.9	2.8^c^ ± 0.2	133.7^b^ ± 5.3	ND	++
Ni	100	29.1^d^ ± 1.4	ND	33.2^c^ ± 1.8	2.5^c^ ± 0.2	124.4^c^ ± 4.4	ND	++
	200	28.7^e^ ± 1.6	ND	30.7^e^ ± 1.2	2.3^d^ ± 0.2	120.0^d^ ± 6.8	ND	++
	400	27.5^f^ ± 1.2	ND	29.1^f^ ± 2.3	2.3^d^ ± 0.2	114.3^e^ ± 7.3	ND	+
	25	35.5^b^ ± 1.7	ND	33.9^c^ ± 1.5	3.2^b^ ± 0.2	140.9^b^ ± 7.5	ND	++
	50	31.6^c^ ± 1.2	ND	31.4^d^ ± 0.6	1.8^e^ ± 0.1	124.2^c^ ± 2.5	ND	++
Pb	100	26.3^d^ ± 0.9	ND	29.2^f^ ± 0.5	1.6^e^ ± 0.1	123.8^c^ ± 3.1	ND	+
	200	23.9^e^ ± 1.2	ND	27.4^g^ ± 0.9	1.5^f^ ± 0.5	121.2^d^ ± 1.8	ND	+
	400	21.5^f^ ± 1.1	ND	26.3^g^ ± 0.6	1.3^f^ ± 0.1	109.8^e^ ± 4.0	ND	+
LSD		1.6		2.4	0.3	8.2		
*F*-value (treatment)		226.0		107.6	78.7	95.9		

a IAA indicates indoleacetic acid; HCN and NH_3_ represent hydrogen cyanide and ammonia, respectively; SA and DHBA indicate salicylic acid and 2,3-dihydroxybenzoic acid, respectively; values are mean of three independent replicates. Mean values denoted with different letters are significantly different according to Duncan's multiple range test (DMRT) at 5% probability level. *IAA production was recorded at 200 μg mL^−1^ concentration of tryptophan. ± indicates standard deviation. ND represents ‘not detected’. LSD is the least significant difference among treatment approaches.

Siderophores, secreted by bacteria,^97^ are yet another metabolically significant class of compounds which chelate iron from the medium and make it inaccessible for uptake by plant pathogens. Hence, by limiting the population of pathogens, siderophores indirectly promote the growth and survival of plants in metal contaminated soils.^98^*Bacillus subtilis* BM2, used in this study, could synthesize small amounts of siderophores under metal free conditions and produced a 11.3 mm zone when grown on CAS agar medium (Table 4). However, disappointingly, the strain did not produce siderophores when grown on CAS agar plates amended with varying doses of Ni and Pb, possibly due to the presence of HDTMA, an essential component of CAS agar medium. In some experiments HDTMA has been found to be inhibitory for microorganisms.^99^ Therefore, HDTMA might have been lethal for strain BM2, ultimately resulting in either death of the bacterial strain or inhibiting the pathways involved in siderophore synthesis. Unlike in solid medium, strain BM2 continued production of siderophores even under metal stress when grown in liquid medium. BM2 synthesized 3.2 μg mL^−1^ 2,3-DHBA and 41.6 μg mL^−1^ salicylate (SA) type phenolates when grown in the presence of 25 μg Ni mL^−1^, which were 11.1% (2,3-DHBA) and 25.5% (SA) less as compared to the untreated control (Table 4). Similarly, Pb at 25 μg mL^−1^ reduced the siderophore production by 11.1% (2,3-DHBA) and 38.1% (SA) relative to the control. Interestingly, the siderophore producing ability of strain BM2 persisted at all concentrations of Ni and Pb although the amounts were comparatively poor at higher doses of each metal. Why siderophore secretion occurred in liquid medium but was completely lost on solid CAS medium is not understood. However, the most probable reason for sustained production of siderophore in liquid medium is the lack of HDTMA in liquid medium.

#### 3.7.2 Production of HCN, ammonia and ACC deaminase

Hydrogen cyanide (HCN), a volatile secondary metabolite released by many bacteria, serves as an important factor that indirectly supports plant development by arresting the growth of pathogens.^100^ Many bacteria are capable of synthesizing HCN even under metal pressure, which could be of immense help to enhance crop production in metal contaminated soils. However, in this study, strain BM2 displayed no signs of HCN production in the presence of varying doses of Ni and Pb, but could secrete HCN when cultured in a metal free environment (Table 4). In agreement with our findings, secretion of HCN by several bacterial genera like *Bacillus* sp., *Pseudomonas* sp. and *Serratia marcescens* has been well documented.^101^ Moreover, the production of ammonia was not significantly affected by varying doses of heavy metals. However, Pb at three concentrations did cause a slight decrease in ammonia production, which was evident from the change in colour intensity of the resulting peptone water after incubation. Also, Ni did not severely affect ammonia production by *B. subtilis* BM2, but did cause a slight reduction in ammonia release at 400 μg Ni mL^−1^ as compared to the metal free control (Table 4).

The ACC deaminase enzyme secreted by many bacteria including *Bacillus* sp.^102^ plays a critical role in lowering the ethylene levels in higher plants, thereby facilitating plant growth even in stressed environments.^103^ Considering this, strain BM2 was tested for its ACC deaminase synthesizing potential. *Bacillus subtilis* BM2 exhibited ACC deaminase activity even in the presence of Ni and Pb, which however, reduced consistently with increasing concentration of Ni and Pb (Table 4). Among the two metals, Pb at 400 μg mL^−1^ demonstrated the maximum reduction in α-ketobutyrate concentration and caused a substantial decline of 47.6% decline in ACC deaminase activity compared to the untreated control. On the other hand, in the presence of 400 μg mL^−1^ of Ni, the concentration of α-ketobutyrate was reduced to 114.3 μmol mg^−1^ protein h^−1^ relative to the fairly large amount (209.5 μmol mg^−1^ protein h^−1^) produced by *B. subtilis* strain BM2 under controlled metal free conditions (Table 4). Interestingly, the continued synthesis of α-ketobutyrate even in the presence of heavy metals suggested the expression of ACC deaminase coding genes present in strain BM2. Such expression for ACC deaminase activity has been previously reported for *Enterobacter* sp. (ACC1 and ACC2) and *Chryseobacterium* sp. (ACC3) under stressed environmental conditions.^104,105^ This distinctive feature of the synthesis of ACC deaminase enzyme by *B. subtilis* BM2 even under metal stress could be useful for enhancing the growth and subsequent production of cereal crops like wheat even in metal contaminated soils, as also reported by other workers.^106^

## 4. Conclusions

The present study primarily focussed on the damaging impact of heavy metals on various biological and chemical characteristics of wheat plants. The metal treated wheat plants unmistakably displayed significant morphological alterations in wheat tissues along with considerable accumulation of heavy metals in plant organs. Also, severe oxidative damage in the wheat plants due to metals was observed, which eventually triggered a strong antioxidant defence response and membrane lipid peroxidation. Moreover, the metal tolerant bacterial strain *Bacillus subtilis* BM2 employed in this study obviated metal toxicity by generating MTs. As a result, *B. subtilis* BM2 was found to be a suitable organism that could serve as an excellent tool for remediation of metal polluted soils. Thus, strain BM2 when applied as an inoculum enhanced the overall performance of wheat by (i) significantly reducing the metal uptake and (ii) lowering the abnormally high levels of proline, antioxidant enzymes and MDA content in plants growing in stressed soils. The ability of strain BM2 to synthesize various active biomolecules like IAA, siderophores, ammonia, and to exhibit ACC deaminase activity even under metal pressure was exceptional. These factors either independently or simultaneously might have played a significant role in enhancing the overall performance of wheat and have provided a valuable insight into why the wheat plants continued to grow and perform even under metal stress. Due to the commendable metal tolerance ability and other distinctive growth promoting features, the metal tolerant *B. subtilis* BM2 could, therefore, serve as a potent bacterial strain, which could be employed in a simple, inexpensive and eco-friendly approach to remediate heavy metal contaminated soils. In summary, due to the ability of *B. subtilis* BM2 to detoxify metals and to efficiently diminish high levels of proline, MDA and antioxidant enzymes in wheat, this microbiological candidate could be targeted for environmental management studies wherein it could efficiently clean up metal contaminated sites and concurrently improve the growth of wheat plants even in metal polluted soils.

## Author contributions

MSK and AZ have conceived and designed the research plan. AR performed the experiments and drafted the manuscript. AZ, AR and BA also contributed in data analysis, discussion and the overall development of manuscript. All authors have read and approved the final version of the manuscript.

## Conflicts of interest

There are no conflicts to declare.

## Supplementary Material
